# Why model?

**DOI:** 10.3389/fphys.2014.00021

**Published:** 2014-01-28

**Authors:** Olaf Wolkenhauer

**Affiliations:** ^1^Department of Systems Biology and Bioinformatics, Institute of Computer Science, University of RostockRostock, Germany; ^2^Stellenbosch Institute for Advanced Study, Wallenberg Research Centre at Stellenbosch UniversityStellenbosch, South Africa

**Keywords:** systems biology, systems medicine, mathematical modeling, cell biology

## Abstract

Next generation sequencing technologies are bringing about a renaissance of mining approaches. A comprehensive picture of the genetic landscape of an individual patient will be useful, for example, to identify groups of patients that do or do not respond to certain therapies. The high expectations may however not be satisfied if the number of patient groups with similar characteristics is going to be very large. I therefore doubt that mining sequence data will give us an understanding of why and when therapies work. For understanding the mechanisms underlying diseases, an alternative approach is to model small networks in quantitative mechanistic detail, to elucidate the role of gene and proteins in dynamically changing the functioning of cells. Here an obvious critique is that these models consider too few components, compared to what might be relevant for any particular cell function. I show here that mining approaches and dynamical systems theory are two ends of a spectrum of methodologies to choose from. Drawing upon personal experience in numerous interdisciplinary collaborations, I provide guidance on how to model by discussing the question “Why model?”

## Why systems biology?

I accepted 10 years ago what was then the first professorship dedicated to “systems biology” in Europe. These 10 years have given me sufficient time to reflect upon the role of mathematical modeling for the study of cellular systems. One particular experience, which I want to share here, is the first encounter of “modelers”, like myself and “experimentalists” in meetings that were set up to discuss the possibility for an interdisciplinary collaboration between our groups.

On such occasions, the question “What is Systems Biology?” is occasionally discussed, although I find the question “Why model?” more important. I shall thus focus on the arguments for mathematical modeling of cellular systems, beginning with my favorite definition of systems biology: Systems biology is the science that studies how biological function emerges from the interactions between the components of living systems and how these emergent properties enable and constrain the behavior of those components^1^. This definition does not only integrate many different views but also highlights the fact that systems biology is not a separate discipline but part of biology and biomedicine. Systems biology is thus an approach to understanding complex, i.e., non-linear spatio-temporal phenomena, across multiple levels of structural and functional organization. And for most projects I have been involved in, this approach is characterized by a combination of experiments with mathematical, statistical, and computational modeling.

From first encounters between my group and colleagues in the biological or biomedical sciences, I learned that even the most enthusiastic experimentalist would harbor the question “Why model?” or “What is it doing for me?” in his/her mind. These are fair questions and I don't find it is easy to answer them in the short time that is typically available in such meetings.

A quick answer is that, for complex systems, all alternatives for understanding the system involve creating a model of some sort. We cannot understand complex systems without modeling. For example, even a diagram that identifies the components of a system (e.g., a network or pathway) and has arrows to indicate interactions between them is a conceptual model. For complex systems, new understanding about how the system might work is generated by transforming one reality into another. This latter, reduced or abstracted form may be a diagram, a physical model or a mathematical model. Many projects in systems biology translate diagrammatic representations into mathematical models. Through simulation, such models help understanding if the original interactions diagram appropriately captured molecular components and interactions related to cell functions (e.g., apoptosis or cell differentiation). The iterative cycle of data-driven modeling and model-driven experimentation, in which alternative hypotheses are postulated and refined until they are validated, helps in identifying new mechanistic details of cell-biological processes and previously unidentified regulatory interactions in the system.

Even though I suggested above that modeling is ubiquitous, the complexity of biological systems and current experimental technological bottlenecks may limit the modeling that can be undertaken in the course of many wet-lab centered research projects. When experimental bottlenecks prevent generating the appropriate data to create a mathematical model that could answer the questions asked in the project, I would emphasize that the modeling process itself requires a way of thinking that can be beneficial to the design of experiments. In the next section, I shall provide some examples to clarify what I mean and convince the skeptical reader of the points made above.

## Examples for the systems biology approach

Earlier, I described meetings in which experimentalists and modelers come together with an interest to collaborate but I have also been in meetings where the value of a systems biology approach and the role of mathematical modeling were questioned. This often leads to a question about “success stories” in systems biology. In response, many people would refer to the virtual heart effort, with Denis Noble as one of its pioneers (Noble, 2008). While the virtual heart project is a truly fantastic example, it is also a long-term effort, involving a large number of researchers. Therefore, this example will not help me convince naysayers in meetings where I sit together with a few colleagues from another faculty across the campus and where we try to identify possibilities for collaborations that may lead to fundable projects in the 3-year projects model that most funding agencies currently use. I shall give here a small selection of examples that I found useful in explaining my colleagues and potential collaborators how modeling can contribute to their work.

The first type of project I like to use as an example, investigates the temporal evolution of cell populations in response to some perturbation. Data from FACS measurements provide information about phenotypic states and a stochastic model allows predicting whether and how subpopulations of cells reach equilibrium proportions over time. These predictions about the expected heterogeneity in the proportions of different tumor cell types over time can be used to design drug-dosing schedules for chemotherapy in cancer research (e.g., Foo and Michor, 2009; Liao et al., 2012). Stochastic models are ideally suited for situations where biological variability as well as uncertainty in measurements may significantly affect the interpretation of data. Unfortunately, the seemingly more abstract notation for stochastic modeling prevents some people from using it. This motivated us to write a textbook on stochastic modeling for systems biology (Ullah and Wolkenhauer, 2011).

The second type of project frequently starts by creating a computerized version of an interaction map that gathers all information about molecular components and interactions that may be relevant to a process under consideration. These maps help us to organize disparate information into a coherent whole. One can then explore the full map and roughly analyze the types of dynamic behavior that it may generate, either by using graph theory or by interpreting interactions as logical relations. Alternatively, one can use the map as the basis for the selection of a subnetwork, which is subsequently investigated with a more detailed kinetic/mechanistic model. For example, based on such a regulatory map of p21, which includes its transcriptional factors, targeting miRNAs and interacting proteins we constructed a kinetic model to describe the repression of the target hub gene p21 by multiple miRNAs. The model was calibrated and validated with northern and western blot data and was subsequently used to predict the effect of different miRNAs expression profiles in combination with their cooperativity on determining the p21 expression levels for different biological contexts (Lai et al., 2012).

The third type of project can be viewed as a mix of the first two types and involves the use of multi-scale models. These allow us to study interactions between molecules, cells, and their environment at different scales, in time *and* space. For example in breast cancer, spatio-temporal models have provided new insights into the progression of the disease by showing how the tumor and stroma interact (e.g., Basanta et al., 2009). The models were used to generate hypothesis about key molecular/cellular interactions that result in a specific formation of cancer cells in their environment. Subsequent microscopy and Western blot experiments confirmed the hypotheses. Multi-scale model often employ agent-based simulations, which are well suited to describe spatio-temporal processes. The behavior of individual “agents” (e.g., cells) can be formulated in terms of a few relatively simple rules, with complex behavior emerging from the interactions among the agents. The two difficulties we have with this approach is that the models are encoded in large amounts of programming code that is often difficult to check and the simulations can also require considerable computational resources. There is thus scope for further improvements of our tools and interesting opportunities for research into the mathematical and computational tools. In another cancer research project, focusing on tumor-stroma interactions, we used this approach to simulate of molecular diffusion in environments with impenetrable barriers. This taught us that modeling requires a careful balance between the simplicity of a model (helping its implementation and efficient simulation) and the required level of detail that makes the model predictive for the application.

Independently of the type of project we undertake, modeling is never its own final goal. It is a tool we use to increase understanding of the biological system and to develop more directed experiments. For example, in one of our longer running collaborations with Robert Jaster, we established a rate equation model describing IFNg induced STAT1 signaling in pancreatic stellate cells. The model was calibrated with experimental time series for STAT1 activation and SOCS1 mRNA expression. Once the model is established, simulations can be used to predict the response of the system to different patterns of stimulation. This allowed us to predict the temporal responses of STAT1 and SOCS1 after a dose split stimulation of the cells with IFNg. These predictions were experimentally validated, reinforcing our confidence that the model was a good starting point for the design of new experiments and emphasizing that the mechanisms described in the model can account for the experimentally observed phenomena (Rateitschak et al., 2010). Such mechanistic modeling of signaling networks can also be used to study differences between cell types. For example, in one of our experiments the time course profiles for phosphorylation and nuclear accumulation of a pathway response showed variations between two cell types. We established a rate equation model and calibrated it with experimental data from both cell types. By using simulations to investigate the variability in the dynamic behavior of the model as a consequence of the uncertainty in the estimated parameter values, we could identify reactions that differ in the two cell types. This provided the basis for a prediction of therapeutic targets that are valid for both cell types (Rateitschak et al., 2012).

Virtually all of the projects in which we have used models, are focused on a particular experimental context, including a chosen experimental model and often linked to specific technologies used to generate data. Each of these projects produces a piece of evidence for or against some hypothesis that is usually addressing a far more general question. For example, many projects that seek a better understanding of apoptosis, will conduct experiments for a relatively small number of relevant components. The results provided by these experiments will then have to be complemented with evidence from other studies to make a plausible argument in the more general context of apoptosis. Review articles in leading journals play an important role in putting the pieces of such puzzles together, helping us to fit a relatively small experiment into a larger picture. This process of “theorizing” in which we integrate and generalize evidence is something I believe should also be supported with mathematical modeling (Wolkenhauer and Green, 2013).

At this stage I hope that the reader is already convinced about the importance of the systems biology approach and usefulness of mathematical modeling to experimental biologists. The good news is that even simple models can often be useful; a detailed model of either a virtual brain or a virtual human is by no means required to have a productive collaboration with an experimental or clinical researcher. One of the most important lessons I learned from working with experimentalists is that the construction of a model promotes a “way of thinking” that critically assesses relevant system variables and their interactions. The process of constructing a model is thus as valuable as the model itself and “modeling as a way of thinking” has a lot more to offer than numerical “predictions”. Mathematical modeling helps us to explain relationships between parts of a system, to illuminate the core dynamics of processes, to bound biological outcomes within plausible ranges, and to illuminate uncertainties (Epstein, 2008). In systems biology, the role of the model is to make something complex intelligible or understandable. To make this contribution, the model should be as simple as possible and as detailed as necessary. In other words, a model should abstract (and thereby reduce) a complex reality into a relatively simple structure. It is through this transformation of one reality into another that we gain understanding. This process however requires us to make assumptions and thus modeling is the *art* of making reasonable assumptions and appropriate choices.

## Navigating through the zoo of modeling approaches

**Figure 1:**
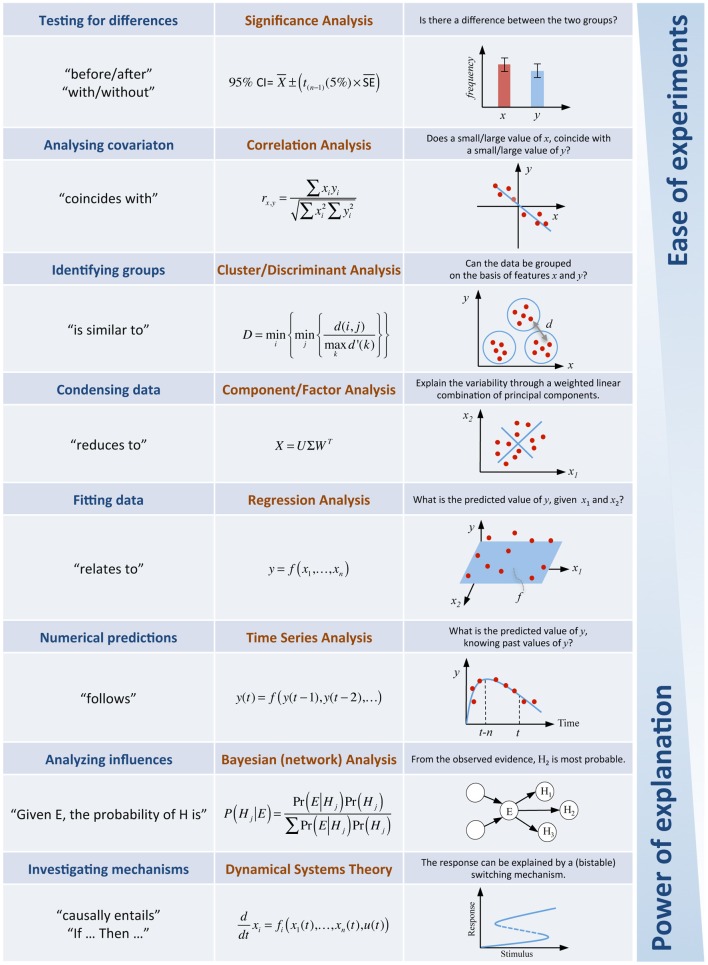
**Answering biological questions through mathematical analyses.** The table illustrates a selection of approaches available in systems biology. In practice, the vast majority of questions in experimental biology concern “differences.” Given that experimental observations vary, statistical testing will establish the significance of a difference. The experiments for this type of question are easy to conduct but little more than establishing a difference is possible. At the other end of the spectrum models of dynamical systems allow investigations about causal mechanisms underlying complex interaction networks. These very powerful explanatory models do however require sufficiently rich quantitative time course experiments, which in many cases are time-consuming, expensive and technically more challenging.

The examples of models described above included stochastic models (specifically Markov processes), rate equation models (ordinary differential equations) and multi-scale models (agent-based simulations). There are many other examples one could give, but instead I will try to summarize what type of frequently used models exist and how they can be used. I do this with the intention of motivating the modeler who reads this essay to formulate his own examples of success stories and to further detail how modeling can be a useful complementary effort for experimental biologists. Figure 1 provides a tabular summary of key questions in biology and illustrates how these can be addressed through mathematical modeling.

There is a whole zoo of modeling approaches to choose from, and which approach is chosen will largely depend upon the type of question asked and on the data available. The vast majority of questions in the life sciences are simply asking about the effect that “differences” have in the biology (e.g., response of patients with or without treatment, phenotypes of wild type vs. mutant, physiological behavior before and after a perturbation). On a very basic level, statistical models in significance testing allow us to determine the significance of, and the uncertainty in, experimentally observed differences. If one is however interested in how changing the levels of a system variable changes the levels of another system variable, this will take us up a notch on the scale of model complexity and move us to correlation analyses. If explicit formulaic relationships between variables are required, then regression models will be an appropriate choice.

However, to address the questions posed in many projects, statistical models are either not sufficient or only the first step toward explanatory models. For if we wish to “explain” the mechanisms underlying some observed phenomenon, mechanistic models are used to analyze interaction networks. The starting point for mechanistic models is usually a network diagram that depicts the components of the system, and uses arrows to indicate the type of interactions that exist between components. For most cases, these interaction maps will involve feedback or feedforward mechanisms, where changes in one variable *y*, will influence another variable *x*, in a way that involve neither direct signaling nor matter conversion between the two. Such regulatory mechanisms can generate non-linear dynamical phenomena, producing counterintuitive observations. If the evidence for the interactions is not strong and the data about the system's behavior is mostly qualitative, one is safer using a logical representation to create models for the analysis of systemic behavior (e.g., a gene being on or off, distinguishing only activation and deactivation etc.). Using this approach one can construct and analyze large networks, if one is able to live with results that are not quantitative. Logical models are often taken as a formal representation and integration of existing knowledge from the literature and are then used to find hints for which system variables may be most important, possibly preparing a more detailed model of a subset of the overall network.

If the relevant components in a network are largely known, one can, for smaller systems, develop detailed parametric models that, when calibrated with experimental data, allow *in silico* experiments to predict the dynamical behavior of the system following perturbations. In this context, sensitivity analysis is an important tool to investigate the system's behavior in response to changes in the system. Such focused mechanistic models represent interactions among system variables in terms of biochemical or biophysical properties (say a rate equation model).

## From systems biology to systems medicine

As research institutions and funding bodies prepare for Systems Medicine, there will be many more meetings in which clinicians and biomedical researchers meet colleagues from engineering, mathematics and computer science in order to discuss the potential for interdisciplinary collaborations. While the use of IT and bioinformatics approaches is perceived as an obvious and valuable addition to projects in a clinical setting, my experience is that mathematical modeling will require more effort to convince your colleagues. At the core of the discussion is the value of methodologies, compared to the enthusiasm about new technologies (Green and Wolkenhauer, 2012; Wolkenhauer et al., 2013).

There is no doubt that advances in the life sciences have been largely driven by new technologies. For example, with next generation sequencing techniques, one notices that the old bioinformatics “mining philosophy” has regained strength. Detecting patterns in the genetic landscape of samples from individual patients will provide useful information, for example, to refine the classification of groups. The data analysis is comparatively easy and leads to the creation of ever smaller shoe-boxes in which to put cases. This approach will however not take us very far and at some point the need to “understand” the reasons behind these pattern and patient groups will resurface.

Returning to the setting I described above, in a first encounter with experimentally working biologists, what does a collaboration with a modeler imply? I suppose, in most cases, the honest answer is “more work”. This is true whether the experimentalist uses a statistical model to explain data, requiring replicates to quantify uncertainty, or a mechanistic model, requiring sufficiently rich quantitative time courses. In general, the more interesting the question being asked is, the greater the effort in generating the data will be. Statistical models, used to detect simple differences between samples, may be enough to support basic hypotheses about the involvement of a molecular component in a process. However, if we are to understand the mechanisms underlying cell and tissue function, there is no escape from the theory of dynamical systems, with costly and time consuming experiments to generate suitable datasets. I am however utterly convinced that the effort will be worth it because it will lead to new ways of thinking about biological complexity and it will immensely increase our understanding about how living organisms function. Such goals cannot be achieved without mathematical modeling.

### Conflict of interest statement

The author declares that the research was conducted in the absence of any commercial or financial relationships that could be construed as a potential conflict of interest.

## Footnotes

^1^I heard this definition for the first time from Jan-Hendrik Hofmeyr (University of Stellenbosch).
